# Dual-signal readout paper-based wearable biosensor with a 3D origami structure for multiplexed analyte detection in sweat

**DOI:** 10.1038/s41378-023-00514-2

**Published:** 2023-03-28

**Authors:** Yuemeng Cheng, Shaoqing Feng, Qihong Ning, Tangan Li, Hao Xu, Qingwen Sun, Daxiang Cui, Kan Wang

**Affiliations:** 1 School of Sensing Science and Engineering, School of Electronic Information and Electrical Engineering, Shanghai Jiao Tong University, Key Laboratory of Thin Film and Microfabrication Technology (Ministry of Education), 200240 Shanghai, China; 2 Department of Plastic and Reconstructive Surgery, Shanghai Ninth People’s Hospital, Shanghai JiaoTong University School of Medicine, 200011 Shanghai, China; 3 School of Naval Architecture, Ocean & Civil Engineering, Shanghai Jiao Tong University, 200240 Shanghai, China

**Keywords:** Chemistry, Engineering

## Abstract

In this research, we design and implement a small, convenient, and noninvasive paper-based microfluidic sweat sensor that can simultaneously detect multiple key biomarkers in human sweat. The origami structure of the chip includes colorimetric and electrochemical sensing regions. Different colorimetric sensing regions are modified with specific chromogenic reagents to selectively identify glucose, lactate, uric acid, and magnesium ions in sweat, as well as the pH value. The regions of electrochemical sensing detect cortisol in sweat by molecular imprinting. The entire chip is composed of hydrophilically and hydrophobically treated filter paper, and 3D microfluidic channels are constructed by using folding paper. The thread-based channels formed after the hydrophilic and hydrophobic modifications are used to control the rate of sweat flow, which in turn can be used to control the sequence of reactions in the differently developing colored regions to ensure that signals of the best color can be captured simultaneously by the colorimetric sensing regions. Finally, the results of on-body experiments verify the reliability of the proposed sweat sensor and its potential for the noninvasive identification of a variety of sweat biomarkers.

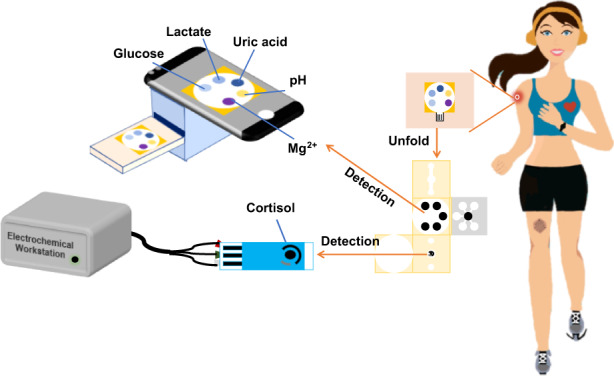

## Introduction

Point-of-care testing (POCT) has developed rapidly in recent years, as it has the advantage of not requiring pretreatment for the analysis of bodily fluids^1–3^. Among POCT devices, wearable sensors, a popular research topic, can be used to measure physicochemical parameters, such as body temperature^4^, electromyography^5^, heart rate^6^, and blood glucose^7^. They can also be simultaneously used for the noninvasive detection of biological fluids, such as human sweat^8,9^, saliva^10,11^, blood^12,13^, urine^14,15^, and tears^16,17^. Of these, sweat can be collected noninvasively and continuously as the subject exercises^1,18^ or through stimulation with pilocarpine^19^. Therefore, wearable sensors are suitable for sweat detection. Sweat contains many chemicals, including water, metabolites (glucose^20,21^, uric acid^22,23^, lactate^24,25^, cortisol^19,26^, and ethanol^27^), electrolytes^28,29^, and macromolecules^30^. These chemicals are closely associated with a wide range of diseases. Glucose detection can be a new method for the noninvasive detection of diabetes^31,32^. Lactate is an important biomarker of fatigue and anaerobic metabolism^33^. High concentrations of uric acid can lead to diseases such as diabetes, hypertension and gout^22^. The pH of sweat can be used to diagnose metabolic alkalosis^34^. Magnesium ions affect the “channels” through which potassium, sodium, and calcium ions move inside and outside cells. Cortisol is associated with stress, circadian rhythms, and major depression^26,35^. Of these, glucose, lactate and uric acid are associated with common physiological disorders, while H^+^, Mg^2+^ and cortisol concentrations are biochemical indicators for assessing mental stress.

Current research on sweat detection can be divided into methods based on electrochemical sensing and optical sensing^36–38^. Colorimetric and electrochemical methods are the most commonly studied. Colorimetric methods for sweat detection^39–41^ are based on the relationship between the concentration of the analyte and the Red–Green–Blue (RGB) intensity of color. By designing microfluidic channels to collect and store the colorimetric sensing-based response of sweat markers, a smartphone can provide a convenient visual readout of the target biomarker^42^. Electrochemical sweat detection involves immobilizing biologically sensitive substances (antibodies^26^, enzymes^1,43^, etc.) on electrodes to generate specific reactions.

With the development of flexible materials and electronics, wearable sensors have been further developed to greatly facilitate sweat detection^6,9,29^. Different materials (polymers^8,43^, paper-based materials^40,44,45^, and hydrogels^18^) and technologies (screen-printed technology^46^, 3D printing technology^47^) have been used to develop wearable sweat sensors. Wearable sweat sensors still have some shortcomings. First, the uniformity of the final color has a large impact on the results of color detection, and the colorimetric signals are easily influenced by light, distance, and angle. Second, the optimal response times of different analytical targets are different such that the optimal colorimetric signal cannot be simultaneously captured. Finally, different sweat analytes are detected in different ways and thus require different detection methods.

In this study, we design and implement a low-cost, patch- and paper-based microfluidic sweat sensor that is easy to use. The sweat sensor simultaneously enables the combined noninvasive and on-body detection of multiple substances in human sweat, including glucose, lactate, uric acid, magnesium ions, and cortisol, as well as the corresponding pH value. The chip has a 3D origami structure to chemically construct hydrophobic microfluidic channels to selectively detect and quantify six analytes in sweat through different sensing strategies with respect to enzymatic reactions, pH indicators, complexes, and molecularly imprinted polymers (MIPs) on the surface of colorimetric and electrochemical electrodes. Wax dams are used to modify the hydrophilic properties of the thread-based channels, control the sequence of reactions of the colorimetric sensors to ensure optimal responses, and thus obtain the best results of detection. Finally, we perform on-body testing to evaluate the proposed sweat sensor, and the results verify its consistency and reliability. It has significant potential for use in the noninvasive detection of multiple biomarkers in sweat.

## Results and discussion

### Principle of reaction

**Fig. 1:**
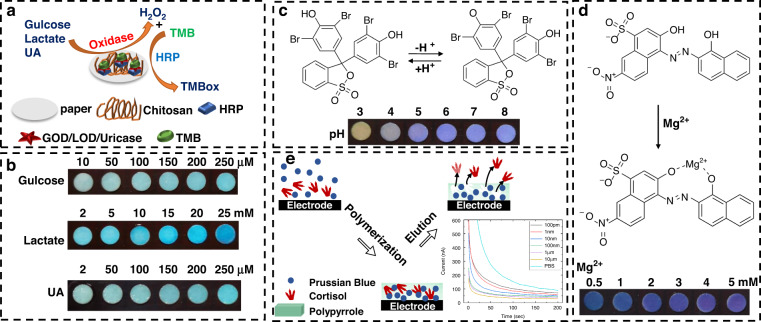
Reaction principles of the six biomarkers in sweat. **a** Glucose, lactate and uric acid sensors respond to the oxidase/HRP cascade reaction. **b** Color change of the glucose, lactate and uric acid sensors at different concentrations. **c** pH sensor using a pH indicator (bromophenol blue as an example). **d** Magnesium ion sensor based on EBT. **e** Schematic diagram of the MIP-based cortisol sensing sensor

Sweat entered the colorimetric sensing area and the electrode layer, and the reaction ensued. The mechanism is shown in Fig. 1. The sensors for glucose, lactate, and uric acid (Fig. 1a, b) were dependent on the corresponding oxidase/horseradish peroxidase (HRP) cascade reaction. The measured indicators in sweat generated hydrogen peroxide (H_2_O_2_) under the catalysis of HRP. H_2_O_2_ oxidized 3,3’,5,5’-tetramethylbenzidine (TMB) and produced blue TMBox. The pH sensors (Fig. 1c) used pH indicators (litmus and bromophenol blue) that responded to changes in pH values ranging from 3 to 8 by changing color from yellow to purple. For sensors of the magnesium ion (Fig. 1d), a chromium black T indicator was used to interact with magnesium and change color from blue to purple. The cortisol electrochemical sensor (Fig. 1e) relied on the selective binding of cortisol to polypyrrole (PPy), thereby blocking electron transfer from the embedded Prussian blue (PB) redox probe. The binding of cortisol and PPy was determined by the cortisol concentration in sweat to allow the quantitative analysis of cortisol.

### Optimization and characterization of sweat chips

**Fig. 2:**
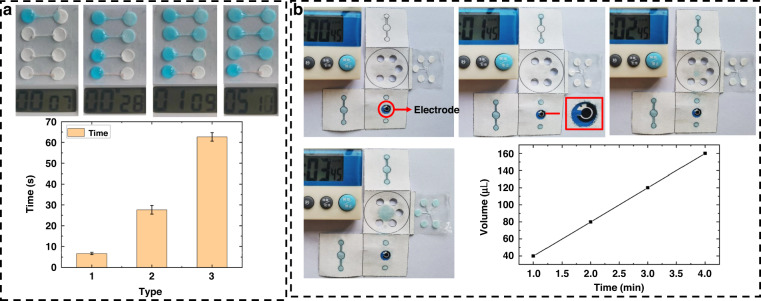
Effect of paper-based microfluidic channels investigated using a Brilliant Blue solution to simulate sweat. **a** Effects of threads soaked in wax for different durations on the duration of liquid flow. **b** Effect of the hydrophilic channel of the 3D paper-based microfluidic chip

The SEM results showed that as the duration in which wax was soaked in sweat increased, wax dams accumulated on the surface of the fiber in the cotton thread channels and thickened, as shown in Fig. 5g, h. With the gradual increase in the hydrophobicity of the cotton thread, the time needed for sweat to flow through increased. We used a bright blue solution to simulate sweat to investigate the effects of threads soaked in wax for different durations on the duration of liquid flow. As shown in Fig. 2a, the end of the thread was fixed in the circular paper-based zone of the reaction. Bright blue drops were added to the reaction zone at one end, and their real-time duration and process of flow were recorded. The results showed that the liquid could pass through the thread within 5 s without the latter being infiltrated by the wax solution. The tip of the cotton thread was briefly dipped in the wax solution, and the liquid could pass through ~25 s after diffusion. The tip of the cotton thread was then dipped in the wax solution until it had been completely infiltrated, and the liquid could then pass through in approximately 1 min. Following this, the cotton thread was completely infiltrated by the wax solution such that the liquid did not pass through it at all. Threads with different flow rates could thus be constructed for applications to control the sequence of the reaction in each area, according to the reaction rates of the target analytes in the five areas of colorimetric reaction, to achieve the best results.

We prepared paper-based microfluidic chips by using the stamping method, which is easy, inexpensive, and fast. The chips had good hydrophobic properties, as needed, and this was confirmed by contact angle analysis. To verify the effect of the hydrophilic channel of the 3D paper-based microfluidic chip, a Brilliant Blue solution was added to its collection layer to observe the mobility of the liquid. Four chips were prepared, and 10 μL of the Brilliant Blue solution was added to them every 15 s. After dripping the solution on them 4, 8, 12, and 16 times from left to right, we unfolded the chips to observe the liquid flow inside. As shown in Fig. 2b, as the volume of the bright blue solution increased, the liquid gradually penetrated the collection layer and entered into the vertical channel, where it first came into contact with the electrode. The sweat then entered the horizontal channel, flowed through the thread into the colorimetric sensing layer, and eventually entered the volatile sweat layer to accumulate and evaporate when the volume of liquid was sufficiently large. The minimum amount of liquid required for the entire experiment was 160 μL.

### Performance of the sweat chip

Images of the colorimetric sensor were acquired by a smartphone, and the intensity of the sensor was correlated with changes in the R, G, and B values to calculate the concentration of the analyte in sweat. The selection of the RGB data channel, the environment for photography, and the optimal response time all influenced the results. As shown in Supplementary Figs. S1–S5a in the ESM, the value of the R channel better fitted the change in color from white to blue caused by enzymatic reactions and that from blue to purple caused by the production of colored complexes. Therefore, we chose values of R for glucose, lactate, uric acid, and magnesium ions to quantify the intensity of color. For artificial sweat in the range of pH = 3–8, the (R + G + B)/3 value provided a significant difference among the pH indicators. The smartphone photo device was designed to ensure a dark environment while the flash of the smartphone served as the light source. Using glucose as an example, the calibration curves of the three were compared under natural light, incandescent light, and light from the photography device. The results (Supplementary Figs. S1–S5b) showed that the curves obtained using the photo device had the best linearity. To determine the optimum reaction time, artificial sweat containing 200 μM glucose, 20 mM lactate, 200 μM uric acid, and 5 mM magnesium chloride at pH = 3 was added to the colorimetric paper-based sensor. The intensities of the colors of glucose, uric acid, and pH were optimal after ~15 min, while those of lactate and magnesium were optimal after ~10 min (Supplementary Figs. S1–S5c). Owing to the fast reactions of lactate and magnesium, more hydrophobic thread channels were used for these biomarkers to delay the flow of sweat into the two sensing regions. Sweat flowed preferentially into the unwaxed thread during the reactions of glucose, uric acid, and pH. Thus, the more hydrophobic the thread used as the channel for lactate and magnesium was, the longer their response times, and this ultimately ensured optimal responses that yielded the best results for the five colorimetric reaction zones. In addition, when the volume of sweat was too large during the experiment, the pH indicator flowed back after completing the reaction and interfered with the detection of the other markers. We applied a hydrophobic thread to the pH channel to prevent this from occurring and obtained satisfactory results for the reaction.

**Fig. 3:**
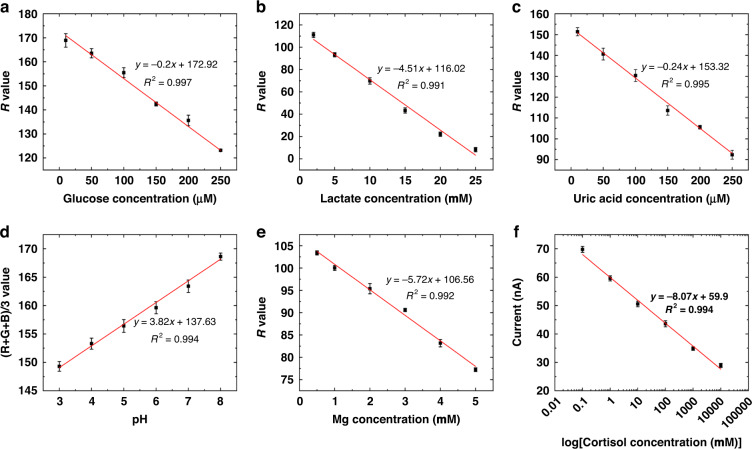
Dependence of the analyzed signals on **a** glucose; **b** lactate; **c** uric acid; **d** pH; **e** magnesium ion; and **f** cortisol. Error bars indicate the standard deviation of the three sensors

The experimental conditions (choice of RGB values, reaction times, reagent concentrations, etc.) were studied and optimized for each assay target by using artificial sweat samples, and details of the optimization and the results are provided in Supplementary Figs. S1–S5 in the ESM. The optimization of the concentrations of HRP, oxidase, TMB, and the indicator led to functionalized, modified filter paper. The dependence of the colorimetric signals on the concentrations of glucose, lactate, uric acid, pH, and magnesium ions under optimal conditions is shown in Fig. 3. The *R*^2^ values of glucose, lactate, uric acid, pH, and magnesium ions were 0.997, 0.991, 0.995, 0.994, and 0.992, respectively.

The MIP electrochemical cortisol sensor allowed quantitative measurement because different concentrations of cortisol molecules occupied the MIP cavity and impeded the charge transfer of Prussian blue. The regression equation for the range of concentrations from 1 × 10^−9 ^M to 10 × 10^−6 ^M was obtained by the concurrent method, with an *R*^2^ of 0.994 for 5 μL of cortisol added to the modified working electrode (Fig. 3f). In contrast, the curve did not change significantly after the dropwise addition of cortisol to the nonimprinted PPy electrode, demonstrating the lack of a cortisol-binding cavity within the PPy layer for detecting cortisol. Supplementary Fig. S6d shows the results of the incubation time optimization for cortisol. When the incubation time was less than 10 min, cortisol within the site was not fully bound. When the incubation time was longer than 10 min, the current response remained largely unchanged. Therefore, 10 min was used as the incubation time for cortisol.

In addition, we investigated whether there was mutual interference among the six biomarkers. The responses of glucose, lactate, uric acid and cortisol were lowest in the corresponding sensor, while the responses of pH and magnesium ions were highest in the corresponding sensor. The results (in Supplementary Figs. S1–3g and S4–6e) showed good selectivity with no mutual interference. As the sweat chips were disposable, their reproducibility was assessed by using five sweat chips to measure the same samples under the same experimental conditions. The results (Supplementary Figs. S1–3h, S4–5f, and Supplementary Fig. S6c) showed that there was no significant difference in the color signals produced by the five biomarkers and that the results of the cortisol sensor were consistent as well.

### Assay of human sweat biomarkers

**Fig. 4:**
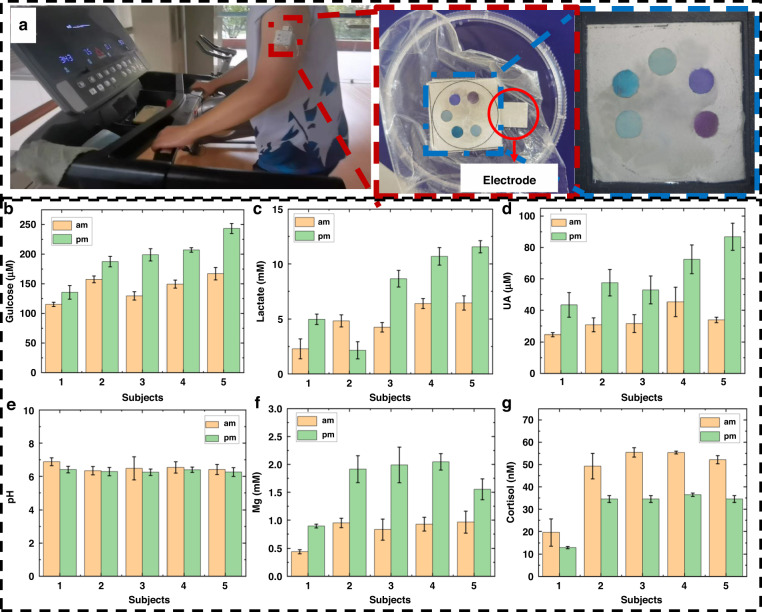
Measurement of sweat biomarker levels in five adult volunteers using sweat sensors. **a** Assay of human sweat biomarkers. **b** Glucose; **c** lactate; **d** uric acid; **e** pH; **f** magnesium ion; **g** cortisol. Test subjects were told to perform low-intensity exercise (treadmill, 5 km/h) at 9 am on an empty stomach. At 6 pm, one hour after eating (carbohydrates, animal offal, hazelnuts, etc.), they performed high-intensity exercise (treadmill, 8 km/h). Test Subject 1 did not exercise, and resting sweat was collected. Error bars indicate the standard deviation of the three sensors

Sweat samples from five adult volunteers in two states were analyzed to simultaneously identify the pH as well as the five biomarkers by using the proposed method, as shown in Fig. 4a. In this case, sweat from Subject 1 was collected while they were in a normal walking state, and that from Subjects 2–5 was collected as they were exercising. In the case of Subject 1, sweat was measured by fixing the chip to their arm for ~75 min. This sensing interval was very long. The performance of the sweat chip was assessed in terms of measuring the amounts of glucose, uric acid, and magnesium ions in the sweat of the subjects based on changes in their state before and after they had eaten (carbohydrates, animal offal high in purines, hazelnuts high in magnesium, and seafood). As shown in Fig. 4b, d, f, the concentrations of all three biomarkers increased after the subjects had eaten, with a consistent trend. However, owing to the different levels of digestion and metabolism of the subjects, the degree of improvement in these markers varied. The concentrations of the biomarkers in the sweat of Subject 1 were lower than those in the sweat of the other subjects, possibly owing to the lack of exercise that resulted in lower metabolism than that of the other subjects.

The performance of the lactate sensor was assessed based on the intensity of the exercise performed by the subjects. Subject 1 had a lower resting lactate level (their *R* value increased) than that produced by exercise, demonstrating that exercise produces lactate. The other volunteers ran on a treadmill at 5 km/h at 9 am and 8–9 km/h at 6 pm. With the increase in the intensity of exercise, the lactate concentration increased to varying degrees (the *R* value decreased). Subject 2 had a high lactate concentration after exercise because they had a sweaty body type. The colorimetric sensor turned yellow, indicating an increase in the *R* value.

The performance of the cortisol sensor was assessed by monitoring changes in the cortisol levels of the subjects in the morning and the afternoon. Figure 4g shows the signals obtained from all five subjects. They were higher in the afternoon than in the morning. The cortisol levels in the sweat of all subjects decreased consistently, demonstrating a high cortisol concentration in the morning and a low concentration in the afternoon. As cortisol levels are related to the level of stress, the extent of cortisol decline varied among the subjects. In addition, exercise also led to an increase in cortisol content, and thus, the resting subject had lower cortisol levels than the other subjects.

## Conclusion

The paper-based microfluidic patch sweat sensor designed and implemented in this study enables the noninvasive and combined on-body detection of multiple indicators. Colorimetric and electrochemical detection was achieved by collecting sweat and analyzing the sweat indicators using different methods. The sensor has a 3D origami structure and is small, inexpensive, and portable. This makes it suitable for disposable use. We also constructed thread-based channels with different hydrophobic properties and used them to control the sequential response time of the colorimetric sensor for the optimal detection of multiple indicators during the same duration of sampling. A matching photo device for the chip ensured the consistency of the conditions of photography each time. The work here verified the potential and versatility of paper-based chips in the noninvasive identification of sweat biomarkers.

## Materials and methods

### Experimental materials and apparatus

Uric acid (UA), magnesium chloride, lactate oxidase (LOD), uricase, 3,3’,5,5’-tetramethylbenzidine (TMB), and Eriochrome Black T Indicator (EBT) were purchased from Macklin Technologies Co., Ltd. (Shanghai, China). Cortisol and glucose oxidase (GOD) were purchased from Shenggong Bioengineering Co., Ltd. (Shanghai, China). L-Lactate was purchased from Bide Medical Technology Co., Ltd. (Shanghai, China), and carboxylated chitosan and peroxidase from horseradish (HRP) were purchased from Innochem Co., Ltd. (Beijing, China). Pyrrole, glucose, and hexane were purchased from Aladdin Co., Ltd. (Shanghai, China), and potassium ferricyanide (K_3_[Fe(CN)_6_]), litmus, bromophenol blue, sodium carbonate (Na_2_CO_3_), filter paper, and absolute ethanol were purchased from Sinopharm Chemical Reagent Co., Ltd. (Shanghai, China). Brilliant Blue was purchased from Myrell Chemical Technology Co., Ltd. (Shanghai, China), alkylenone dimer (AKD) and triethanolamine were purchased from Shaoxin Biotechnology Co., Ltd. (Shanghai, China), phosphate buffer (PBS) was purchased from Jinxin BioReagent Studio, artificial sweat (ISO 3160-2) was purchased from Gao Xin Chemical Glass Instrument Co., Ltd., and cotton thread and wax were purchased from the market. Screen-printed electrodes were purchased from Zensor Electrochemical Cooperative and were modified with a CHI-660D ChenHua electrochemistry workstation. The cotton thread was characterized by scanning electron microscopy (SEM), and the hydrophilic and hydrophobic properties of the surface of the filter paper were characterized by a contact angle analyzer (DSA100).

### Preparation of sweat chips

**Fig. 5:**
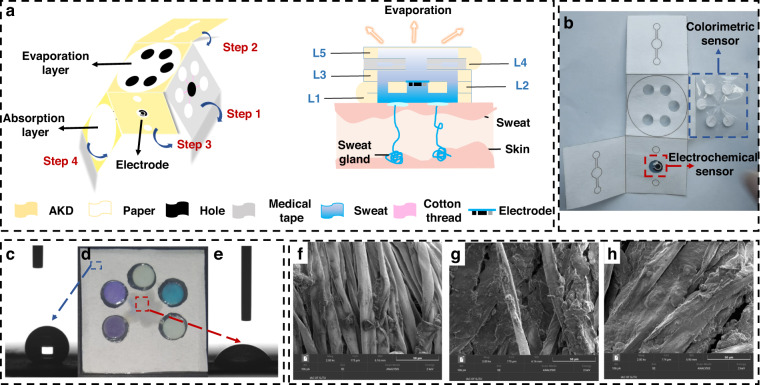
Diagram of the structure of the paper-based microfluidic noninvasive sweat sensor. **a** Diagram of the overall structure of the sweat sensor. **b** Overall solid view of the sweat sensor. **c**–**e** Contact angle tests of the hydrophobic and hydrophilic regions of the paper in the foldable sweat sensor. **f**–**h** SEM diagrams of cotton threads with different degrees of hydrophobicity (**f**: passes completely; **g**: passes for a certain time; **h**: cannot pass)

The wearable sweat chip consisted of two main parts: a microfluidic channel and a sensing area. It was square in shape, and each of its sides was 36 mm long. Its structure is shown in Fig. 5a, b. The chip formed a 3D channel from bottom to top that contained a collection layer (L1), a vertical channel (L2), an electrode layer, a horizontal channel (L3), a colorimetric sensing layer (L4), and a sweat evaporation layer (L5). Screen-printed electrodes were attached to the electrode layer to measure the cortisol in sweat. The colorimetric sensing layer consisted of five 10-mm-long cotton thread-based channels modified by using wax dams of different lengths and a circular sensing area with a diameter of 6 mm (filter paper modified with a specific chromogenic reagent). The remaining design of the layer (shown in Supplementary Fig. S7 in the ESM for paper-based channels) was patterned by using a chemical capping method^48^ to form the desired hydrophilic region. The entire process of sweat flow was as follows: sweat was absorbed through the collection layer, flowed up the vertical channel and the electrode layer through a chromatographic phenomenon, and reacted at the electrode layer. It flowed up again into the lateral channel, gathered at its center, and flowed into the colorimetric sensing layer. Finally, the excess sweat reached the evaporative layer.

The scheme for the preparation of each layer was as follows:

i. Preparation of 3D paper-based microfluidic chips:
  The paper-based microfluidic pattern was designed using CAD. The hydrophilic pattern is represented by the white area in Fig. 5a, and the hydrophobic area formed by AKD is represented by the yellow area. The cut filter paper was first subjected to drying (37 °C in an oven), soaking (6 g/L AKD, the results of different AKD concentrations are shown in Supplementary Fig. S8), and drying again (37 °C oven) to form a clear hydrophobic barrier. Then, stamps of triethanolamine printing oil were used to stamp the corresponding areas, and they were placed in a 105 °C oven to dry to attain patterned hydrophobic areas. The hydrophobicity of the filter paper is shown in Fig. 5c–e.
  Cotton thread was used as the area of the channel. It was soaked in 10 mg/mL of an Na_2_CO_3_ solution (heated in a water bath for 10 min) and anhydrous ethanol (1 h) to remove surface wax and organic matter. The hydrophilic cotton threads were then placed in an oven at 37 °C to dry. The pretreated cotton threads were soaked in a wax solution for different times and then placed on a hot plate at 80 °C to allow the wax solution to diffuse within the cotton threads with different degrees of hydrophobicity. This was used to control the duration of passage of the liquid through the channel. SEM images of the cotton threads are shown in Fig. 5f–h.
ii. Modification of the colorimetric sensing layer:
  We prepared sensors for glucose, lactate, and uric acid by the dropwise addition of chitosan, HRP, the corresponding oxidase, and TMB to the colorimetric sensing region. The volumes of HRP, oxidase, and TMB were modified and optimized. The results showed that when changes in their volumes for the glucose sensor were 0.2 mg/mL, 200 U/mL, and 15 mM, the sensor delivered the best performance. Similarly, the optimum changes in their volumes for the sensors of lactate and uric acid were 0.5 mg/mL, 20 U/mL, and 10 mM and 0.4 mg/mL, 100 U/mL, and 15 mM, respectively. The pH indicator (20:1 mixture of litmus and bromophenol blue) was modified for pH detection. The volume of the chromium black T indicator (1 g/L, 5 μL) was modified to detect magnesium ions. After drying the sensing and channel regions at room temperature, the colorimetric sensing layer was assembled using medical-grade tape and fixed to the 3D paper-based microfluidic chip. This was stored in a refrigerator at 4 °C.
iii. Modification of screen-printed electrodes:
  The MIP films were fabricated by cyclic voltammetry (CV)-based electropolymerization in a PBS solution (0.1 M, pH = 7.4) containing 0.02 M pyrrole, 5 mM FeCl_3_, 5 mM K_3_[Fe (CN)_6_], 6 mM cortisol, and 0.1 M HCI at a potential range of −0.2 V to +0.9 V and a scan rate of 50 mV/s for ten cycles. After the electropolymerization process, the electrode was washed twice with deionized water to remove the remaining compounds. The MIP membrane was subjected to 20 cycles in PBS solution (0.1 M, pH = 7.4) by using CV over a potential range of –0.2 to +0.8 V at a scan rate of 50 mV/s. The embedded cortisol molecules were extracted from the MIP membrane to generate complementary cavities.

The method used for the MIP above was applied to prepare the nonimprinted polymer (NIP), except that the cortisol molecule was not included as a template in the polymerization step.

### Detection of sensing signals

**Scheme 1:**
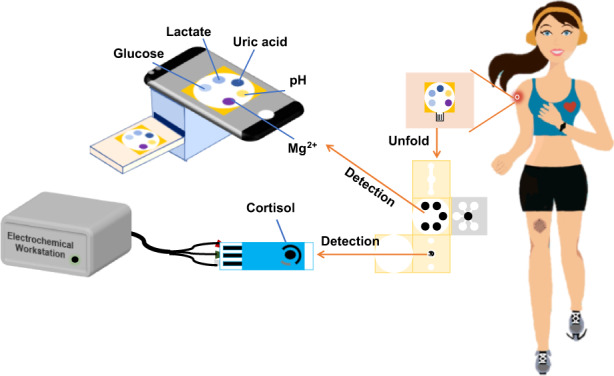
Wearable sweat sensor based on a 3D paper structure for simultaneous analysis of multiple biomarkers

The chip included areas for both colorimetric and electrochemical sensing. Image-related information was captured via a smartphone, and ImageJ was used to analyze and process the color signals. The electrochemical workstation was then used to detect and analyze the electrode signals (Scheme 1).

i. Detection of colorimetric signals:
  A 30 μL sample of artificial sweat was dropped onto the chip. After 30 s, it rapidly entered the colorimetric sensing area via the hydrophobically modified thread-based channel to generate a colorimetric signal. For quantitative analysis, the color signal was collected by using a smartphone (HUAWEI P40 PRO), and the RGB color signal was analyzed by using the color analysis software ImageJ for the fast and accurate detection of the biomarkers.
  To ensure a consistent imaging environment (light, angle, and distance), a smartphone photo device was designed by using SolidWorks software and manufactured by using a 3D printer. The overall size of the device was 158 mm (L) × 72 mm (W) × 90 mm (H). It consisted of a smartphone, a black box, and a loading platform for the chip. The device was made of black resin to avoid interference from ambient light and unwanted reflections of light from inside the device during measurement. The size of the upper surface of the device was determined by the size of the smartphone. The loading platform was used to hold the paper-based chip, and the overall height of the device was determined by the focal length of the camera. It is shown in Supplementary Fig. S9 in the ESM.
ii. Electrochemical signal detection:
  The screen-printed electrodes that were purchased formed a three-electrode system. The working electrode (3 mm in diameter) was a modified carbon electrode, the reference electrode was a Ag electrode, and the counter electrode was a carbon electrode. The electrode substrate was polypropylene, and the electrodes were adhered to the paper-based chip by medical tape. Chrono-current methods were implemented by using 0.1 V (relative to the Ag reference) to obtain calibration curves and implement on-body detection.

### Clinical samples

This study was approved by the Medical Ethics Committee of Shanghai Jiao Tong University, China. All volunteers signed a document providing their informed consent, and all methods were performed in accordance with the relevant guidelines and regulations. The on-body assessment of the microchip was performed on five healthy volunteers (three males and two females). Before the test, the subjects were asked to perform low-intensity exercise (treadmill, 5 km/h) at 9 am on an empty stomach. At 6 pm, one hour after having eaten (carbohydrates, animal offal, hazelnuts, etc.), they performed high-intensity exercise (treadmill, 8–9 km/h). One of the subjects did not exercise, and their resting sweat was collected. The procedure was as follows: (i) The sweat chip was fixed to the subject’s upper-left arm with medical tape. (ii) Half an hour of exercise caused their sweat to reach the sensing area through the microfluidic channel. (iii) Once the response had been recorded, the chip was removed and used for data collection and analysis.

## Supplementary information

SUPPLEMENTAL MATERIAL
