# Supplementary material for: Dual-signal readout paper-based wearable biosensor with a 3D origami structure for multiplexed analyte detection in sweat
Source: Microsyst Nanoeng. 2023 Mar 28;9:36. doi: 10.1038/s41378-023-00514-2 (PMC10042807; doi:10.1038/s41378-023-00514-2)
Supplement: Supplementary file 1 — SUPPLEMENTAL MATERIAL [file 41378_2023_514_MOESM1_ESM.docx]

**Supplementary Material**

**Dual-signal readout paper-based wearable biosensor for multiplexed analyte detection in sweat with 3D origami structure**

Yuemeng Cheng^1,#^, Shaoqing Feng^2,#^, Qihong Ning^1^, Tangan Li^1^, Hao Xu^3^,Qingwen Sun^1^, Daxiang Cui^1^ and Kan Wang^1^*

^1^School of Sensing Science and Engineering, School of Electronic Information and Electrical Engineering, Shanghai Jiao Tong University, Key Laboratory of Thin Film and Microfabrication Technology (Ministry of Education), Shanghai 200240, China.

^2^Department of Plastic and Reconstructive Surgery, Shanghai Ninth People’s Hospital, Shanghai JiaoTong University School of Medicine, Shanghai, 200011, China.

^3^School of Naval Architecture, Ocean & Civil Engineering, Shanghai Jiao Tong University, Shanghai 200240, China.

*Corresponding author: wk_xa@163.com

# Yuemeng Cheng and Shaoqing Feng contributed equally to this work.

Co-authors Email: Yuemeng Cheng: cym1023@163.com

Shaoqing Feng: [fmmufsq@163.com](mailto:fmmufsq@163.com)

Qihong Ning: nqihong_a@sjtu.edu.cn

Tangan Li: [andrewl1234@sjtu.edu.cn](mailto:andrewl1234@sjtu.edu.cn)

Hao Xu: [xudahao@sjtu.edu.cn](mailto:xudahao@sjtu.edu.cn)

Qingwen Sun: wendy1166@163.com

Daxiang Cui: [dxcui@sjtu.edu.cn](mailto:dxcui@sjtu.edu.cn)


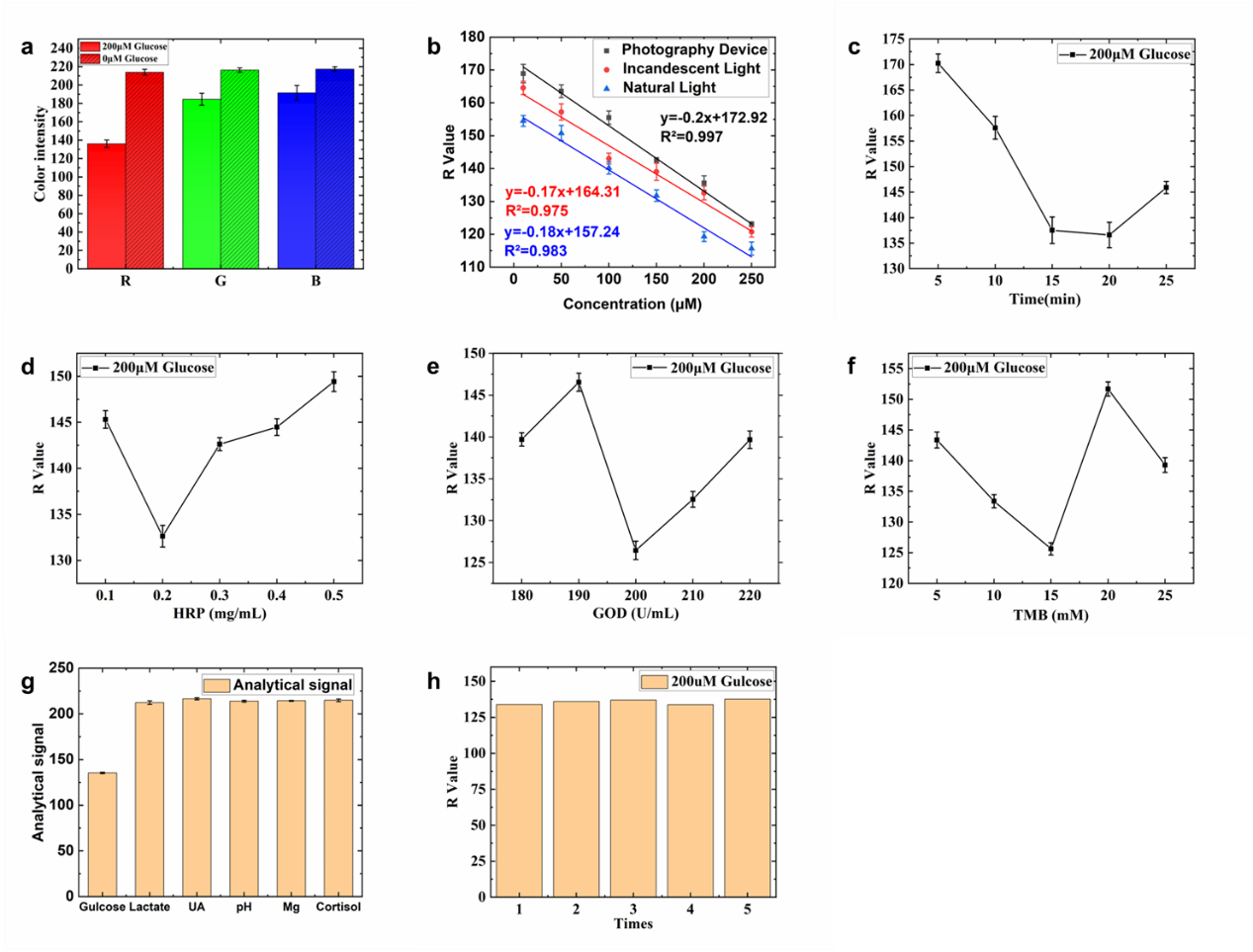


Figure. S1 Optimization of the paper-based glucose sensor. (a) Comparison of R, G and B values corresponding to 0 and 200 μM glucose and selection of the R value. (b) Comparison of the linearity for three lighting conditions: natural light, incandescent light and the photography device, with the best results obtained using the photography device. (c) Using 200 μM glucose, the color was darkest at 15 min in the 0-25 min range. (d) Using 200μM glucose, the best result was obtained when the HRP concentration was 0.2mg/mL. (e) Using 200μM glucose, the best result was obtained when the GOD concentration was 200U/mL. (f) For 200μM glucose, the best effect was achieved when the TMB concentration was 15mM. (g) Biomarkers for the five colorimetric sensors in this paper were tested for mutual interference with each other, it showed good selectivity. (h) With five different sensors, the reproducibility was tested. It showed that there was no statistical difference. Error bars indicate the standard deviation of the 3 sensors.


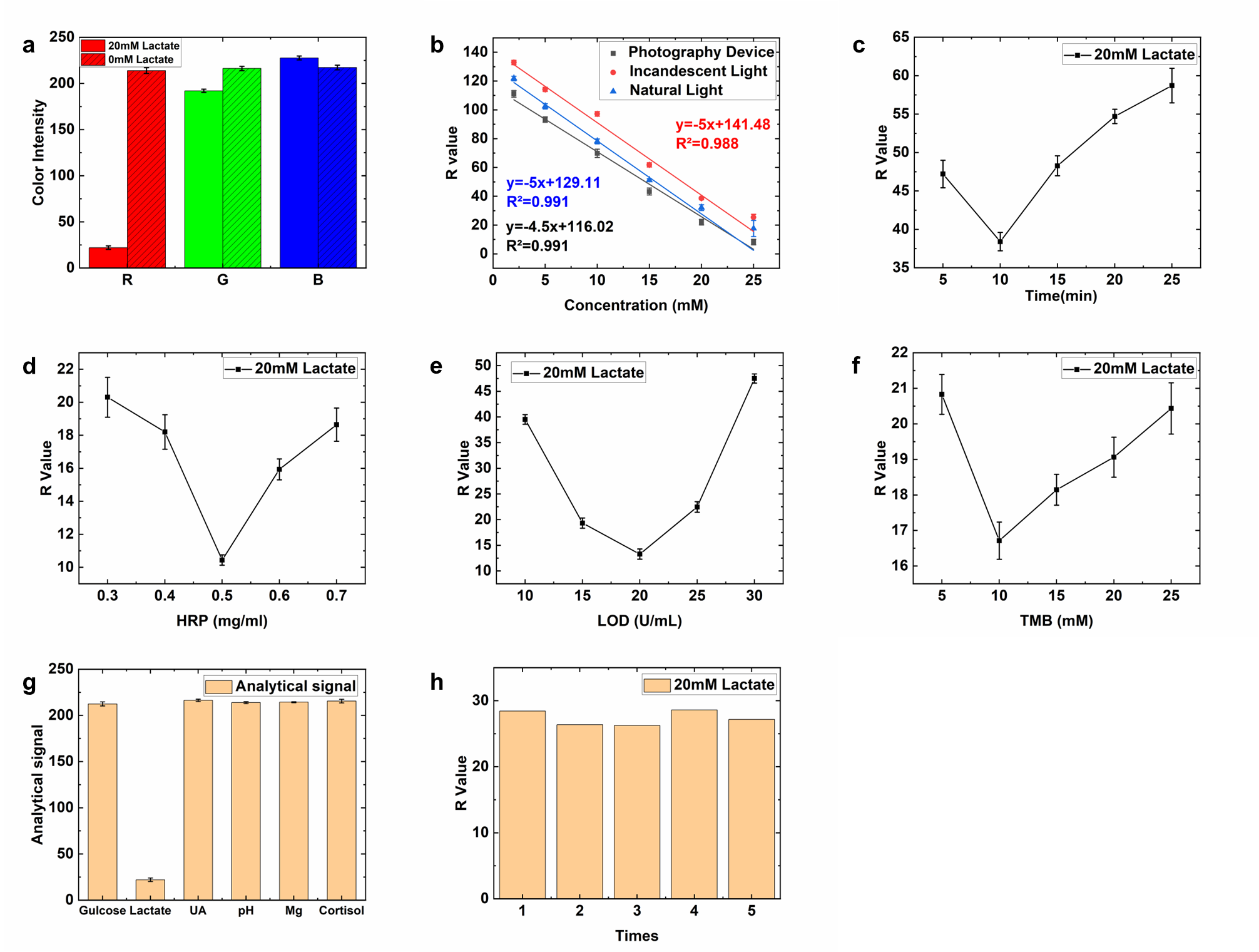


Figure. S2 Optimization of the paper-based lactate sensor. (a) Comparison of R, G and B values corresponding to 0 and 20mM lactate and selection of the R value. (b) Comparison of the linearity for three lighting conditions: natural light, incandescent light and the photography device, with the best results obtained using the photography device. (c) Using 20mM lactate, the color was darkest at 10min in the 0-25min range. (d) Using 20mM lactate, the best result was obtained when the HRP concentration was 0.5mg/mL. (e) Using 20mM lactate, the best result was obtained when the LOD concentration was 20U/mL. (f) For 20mM lactate, the best effect was achieved when the TMB concentration was 10mM. (g) Biomarkers for the five colorimetric sensors in this paper were tested for mutual interference with each other, it showed good selectivity. (h) With five different sensors, the reproducibility was tested. It showed that there was no statistical difference. Error bars indicate the standard deviation of the 3 sensors.


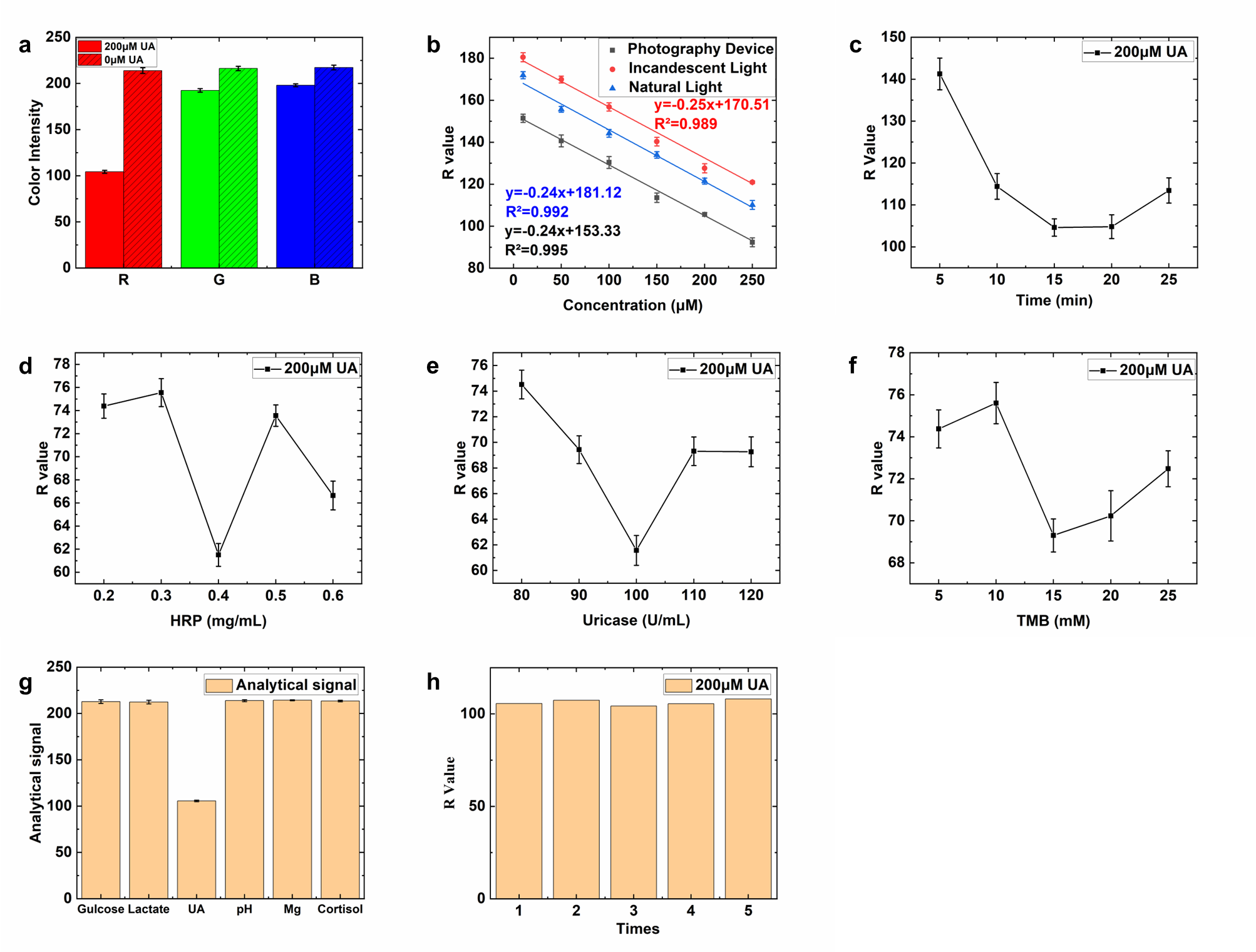


Figure. S3 Optimization of the paper-based uric acid sensor. (a) Comparison of R, G and B values corresponding to 0 and 200 μM uric acid and selection of the R value. (b) Comparison of the linearity for three lighting conditions: natural light, incandescent light and the photography device, with the best results obtained using the photography device. (c) Using 200 μM uric acid, the color was darkest at 15 min in the 0-25 min range. (d) Using 200μM uric acid, the best result was obtained when the HRP concentration was 0.4mg/mL. (e) Using 200μM uric acid, the best result was achieved when the Uricase concentration was 100U/mL. (f) For 200μM uric acid, the best effect was achieved when the TMB concentration was 15mM. (g) Biomarkers for the five colorimetric sensors in this paper were tested for mutual interference with each other, it showed good selectivity. (h) With five different sensors, the reproducibility was tested. It showed that there was no statistical difference. Error bars indicate the standard deviation of the 3 sensors.


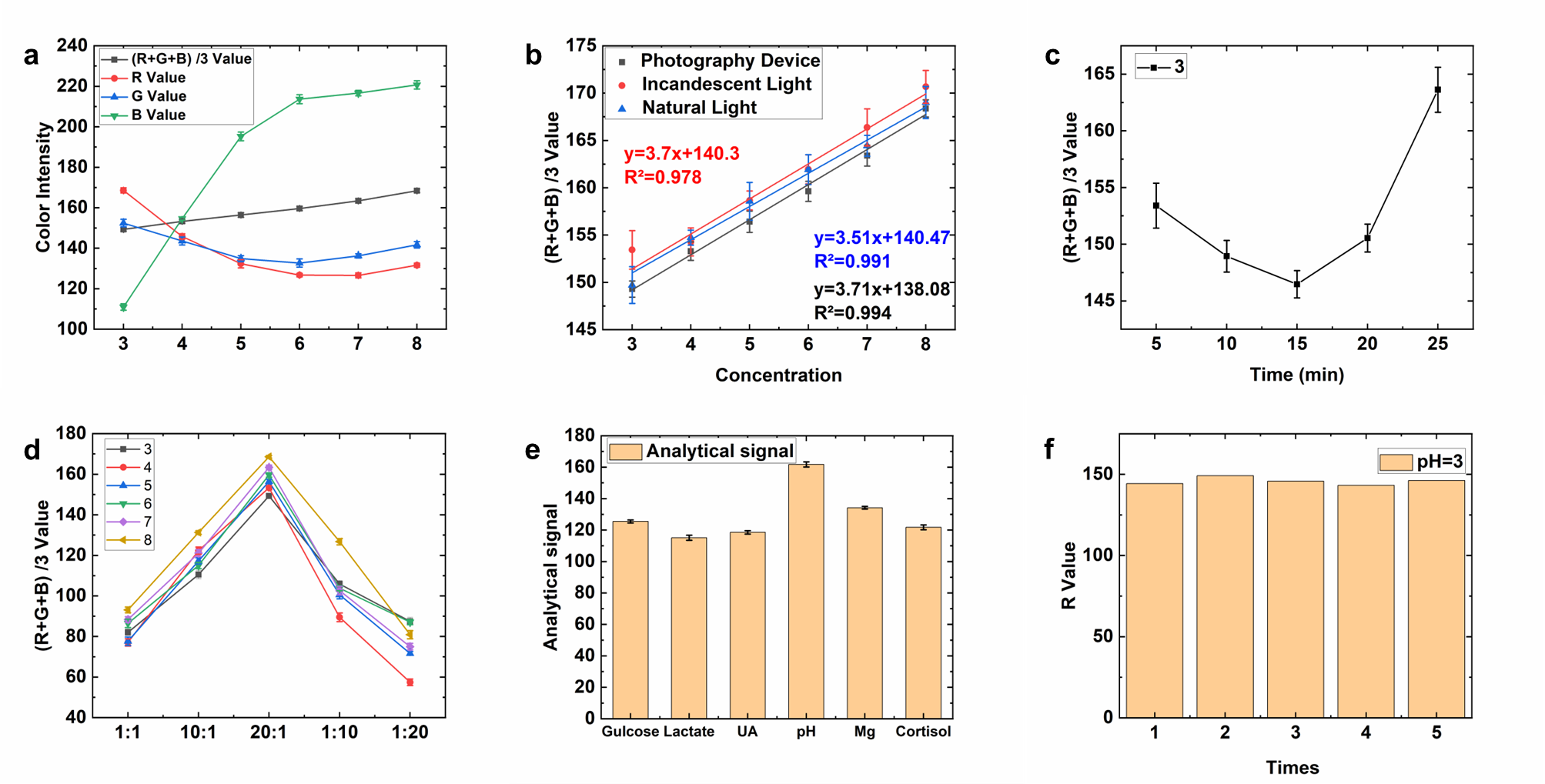


Figure. S4 Optimization of the paper-based pH sensor. (a) Comparison of the linearity of the corresponding R, G, B, (R+G+B)/3 values in the Ph3-8 range, with the (R+G+B)/3 value selected. (b) Comparison of linearity for three lighting conditions: natural light, incandescent light and the photography device, with the best results obtained using the photography device. (c) With pH=3, the color was darkest at 15 min in the 0-25 min range. (d) The ratio of litmus to bromophenol blue was 1:1,10:1,20:1,1:10,1:20 respectively, comparing the corresponding pH and choosing the ratio of 20:1. (e) Biomarkers for the five colorimetric sensors in this paper were tested for mutual interference with each other, it showed good selectivity. (f) With five different sensors, the reproducibility was tested. It showed that there was no statistical difference. Error bars indicate the standard deviation of the 3 sensors.


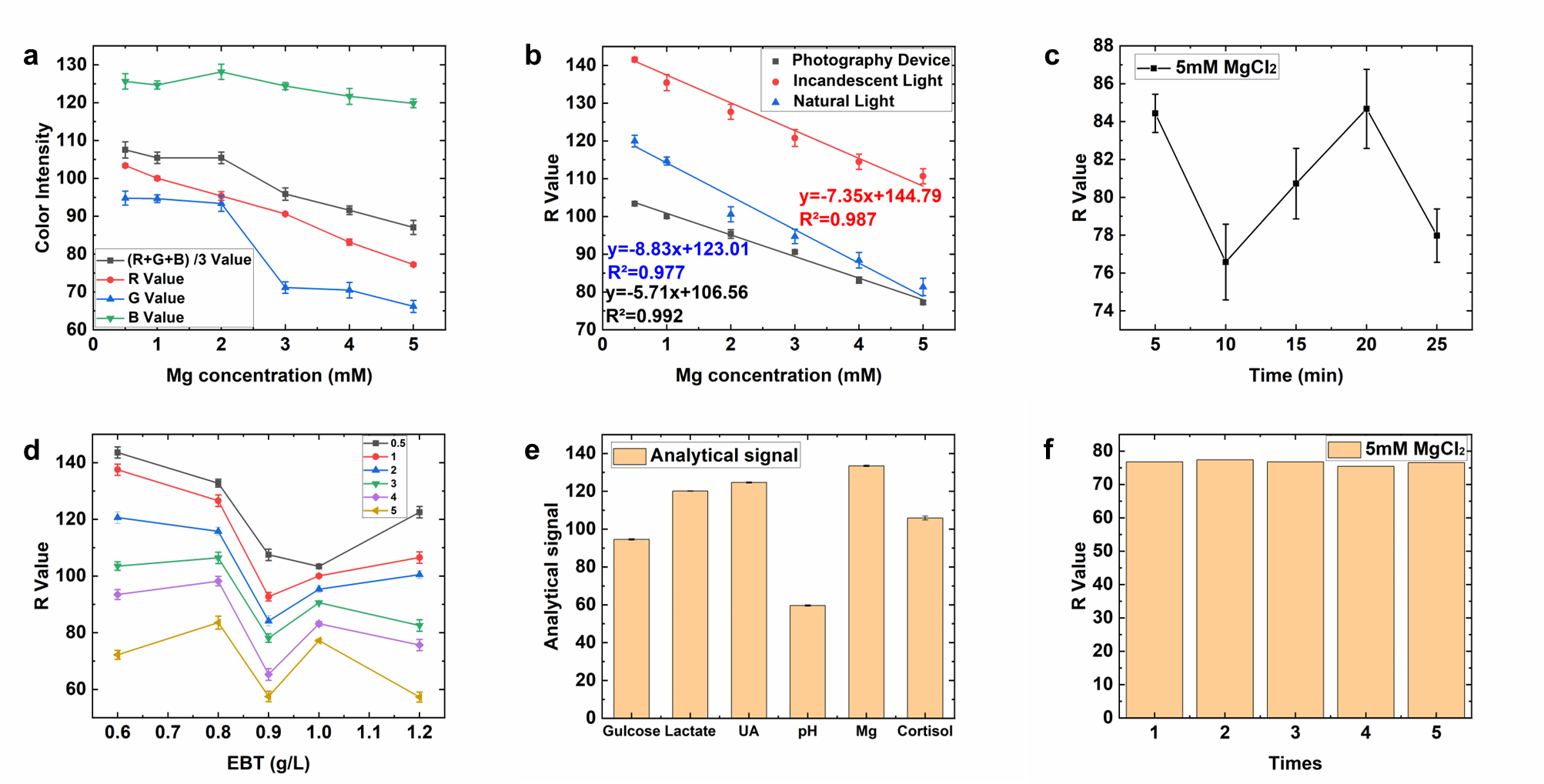


Figure. S5 Optimization of the paper-based magnesium sensor. (a) Comparison of the linearity of the R, G, B, (R+G+B)/3 values corresponding to magnesium ions (0.5-5 mM) and selection of the R value. (b) The linearity was compared for three lighting conditions: natural light, incandescent light and the photography device, with the best results obtained using the photography device. (c) Using 5mM Mg^2+^, the color was darkest at 10min in the 0-25min range. (d) The best result was obtained when the EBT concentration was 1g/L for 5mM Mg^2+^. (e) Biomarkers for the five colorimetric sensors in this paper were tested for mutual interference with each other, it showed good selectivity. (f) With five different sensors, the reproducibility was tested. It showed that there was no statistical difference. Error bars indicate the standard deviation of the 3 sensors.


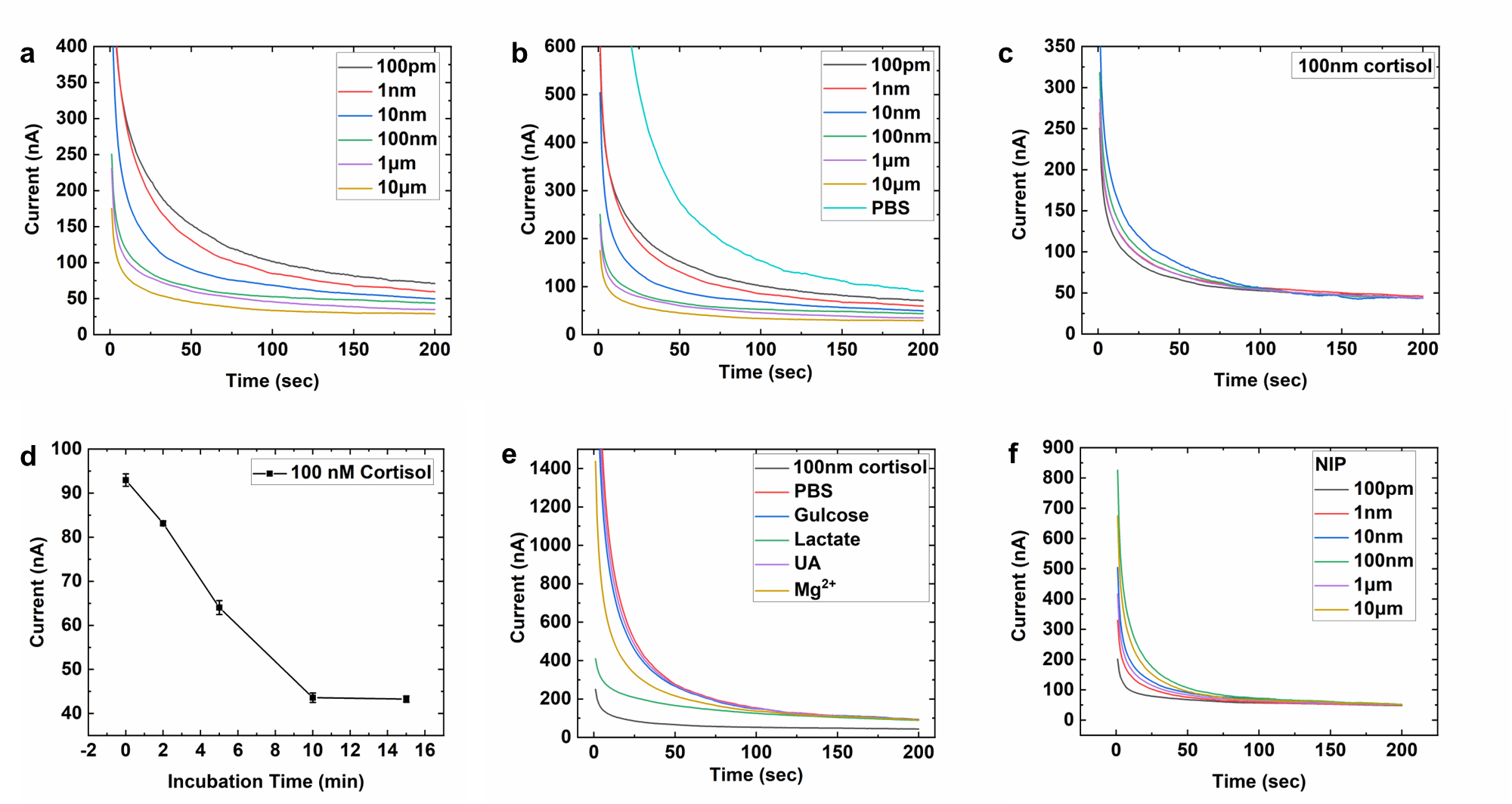


Figure. S6 Electrochemical sensor for cortisol. (a) Electrochemical response of the MIP sensor to different concentrations of cortisol. (b) Electrochemical response of the MIP sensor to different concentrations of cortisol and PBS. (c) The reproducibility of the cortisol sensor was demonstrated by five sensors responding to 100 nM cortisol. (d) Effect of different incubation times on the current response of MIP. (e) The MIP cortisol sensor responded to the addition of glucose, lactate, uric acid and magnesium with no change in response, followed by the addition of 100 nM cortisol with a significant response. (f) Response of the NIP sensor to different concentrations of cortisol, with essentially no change in response to different concentrations of cortisol.


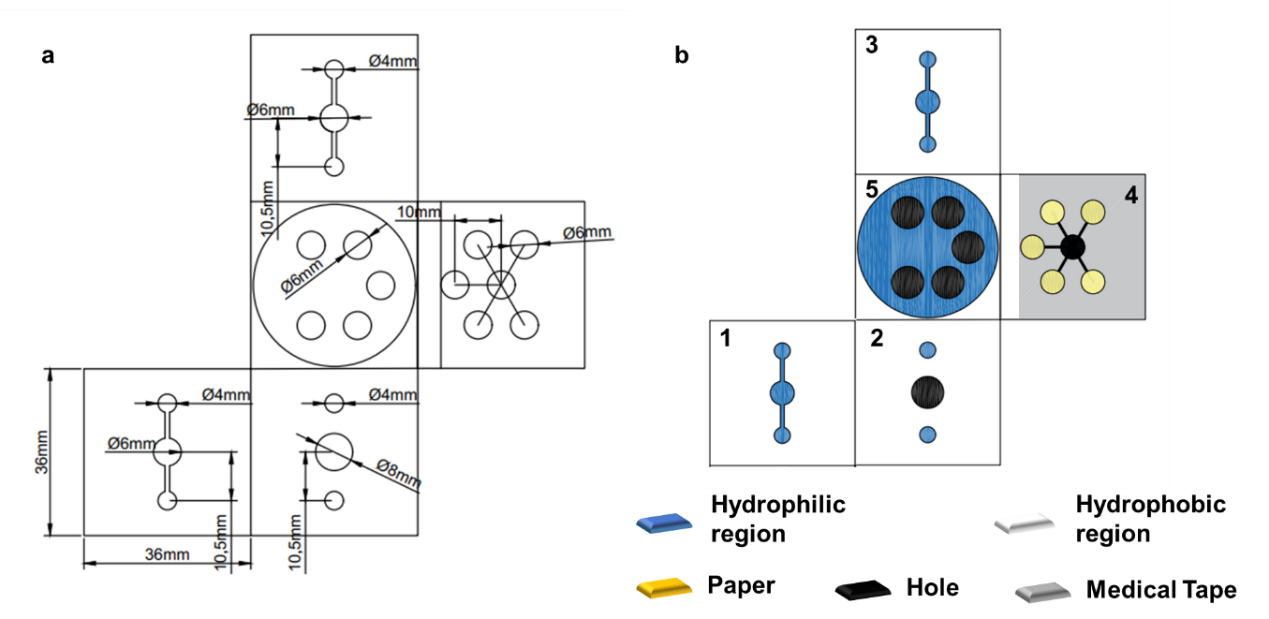


Figure. S7 Paper-based microfluidic channel and sensing area. (a) Dimensional design drawing. (b) functional schematic. The blue part is the hydrophilic area of the filter paper. The white part is the hydrophobic area of the filter paper. The black circular area is the filter paper cut to form holes. The yellow part is the circular colorimetric reaction area. The black straight line is the thread-based for hydrophilic and hydrophobic optimization and the grey part is the medical tape.


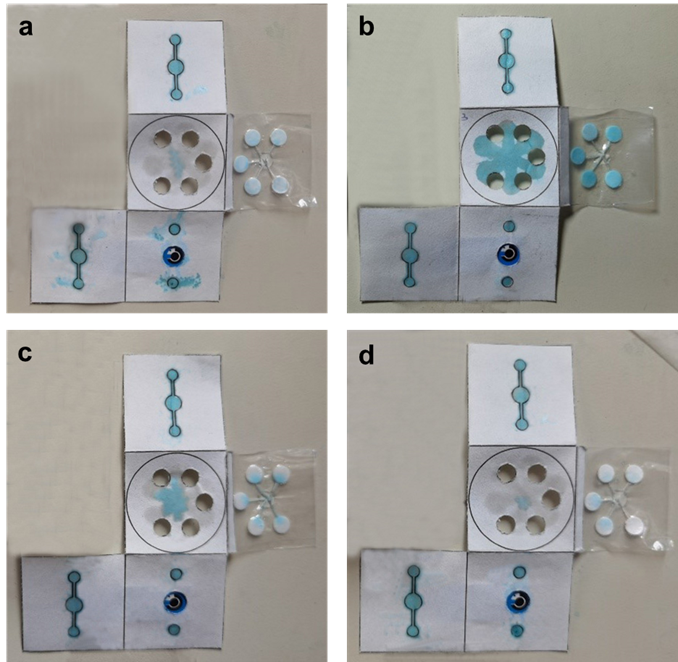


Figure. S8 Effect of different concentrations of AKD solutions on interlayer flow in 3D microfluidic channels. (a) 4.0 g/L. (b) 6.0 g/L. (c) 8.0 g/L. (d) 10.0 g/L. The AKD concentration of 6 g/L was chosen in order to ensure a fast and homogeneous filling of the liquid and to ensure that the filter paper would be able to limit the domain of the solution.


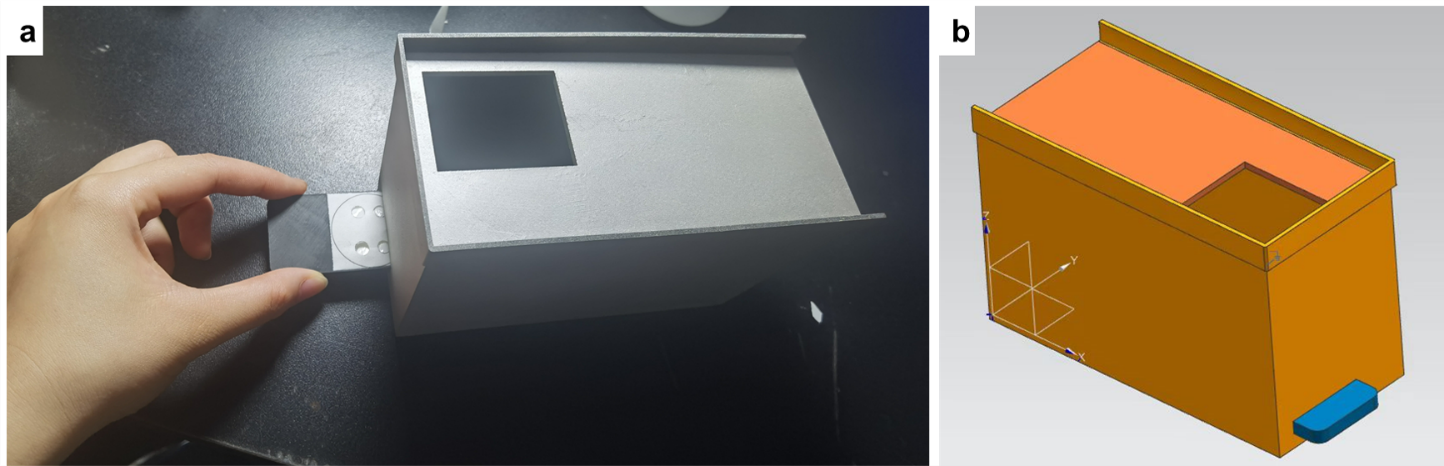


Figure. S9 The smartphone photo device consisted of a black box and a loading platform for the chip. The device was made of black resin to avoid interference from ambient light and light reflection. The size of the upper surface of the device was determined by the size of the smartphone (158 mm (L) × 72 mm (W)), with the position of the camera and flash reserved according to the smartphone (48 mm (L) × 38 mm (W)). The loading platform was used to hold the paper-based chip (50 mm (L) × 38 mm (W) × 6 mm (H)) and the height of the overall device (90 mm (H)) was determined by the focal length of the camera.
